# Application of Data-Centric Supervised Machine Learning to Predict Phenotypic Activity Against Clinically Relevant Stages of *Trypanosoma cruzi*

**DOI:** 10.3390/pharmaceutics17121513

**Published:** 2025-11-23

**Authors:** Nicolás Pérez-Mauad, Lucas N. Alberca, Alejandra C. Schoijet, Salome C. Vilchez Larrea, Emilia M. Barrionuevo, Giuliana Muraca, Valeria Sülsen, Catalina D. Alba-Soto, Guillermo D. Alonso, Alan Talevi

**Affiliations:** 1 Instituto de Química y Metabolismo del Fármaco (IQUIMEFA), CONICET-Universidad de Buenos Aires, Buenos Aires C1113, Argentina; npmauad@gmail.com (N.P.-M.); vsulsen@ffyb.uba.ar (V.S.); 2 Facultad de Farmacia y Bioquímica, Universidad de Buenos Aires, Buenos Aires C1113, Argentina; 3 Laboratorio de Investigación y Desarrollo de Bioactivos (LIDeB), Departamento de Ciencias Biológicas, Facultad de Ciencias Exactas, Universidad Nacional de La Plata, La Plata C1904, Argentina; lucasalberca@gmail.com (L.N.A.); ebarrionuevo@quimica.unlp.edu.ar (E.M.B.); muracagiuliana@gmail.com (G.M.); 4 Signaling and Adaptive Mechanisms in Trypanosomatids Laboratory, Instituto de Investigaciones en Ingeniería Genética y Biología Molecular “Dr. Héctor N. Torres”, Consejo Nacional de Investigaciones Científicas y Técnicas, Buenos Aires C1428, Argentina; aschoijet@gmail.com (A.C.S.); vilchez.ingebi@gmail.com (S.C.V.L.); galonso@dna.uba.ar (G.D.A.); 5 Departamento de Química Biológica, Facultad de Ciencias Exactas y Naturales, Universidad de Buenos Aires, Buenos Aires C1428, Argentina; 6 Departamento de Fisiología, Biología Molecular y Celular, Facultad de Ciencias Exactas y Naturales, Universidad de Buenos Aires, Buenos Aires C1428, Argentina; 7 Instituto de Investigaciones en Microbiología y Parasitología Médica (IMPaM), CONICET-Universidad de Buenos Aires, Buenos Aires C1121, Argentina; catalina.alba@gmail.com

**Keywords:** chagas disease, machine learning, QSAR, phenotypic screening, virtual screening, in silico screening, drug repurposing, *Trypanosoma cruzi*, ensemble learning, data-centric machine learning

## Abstract

**Background/Objectives**: Chagas disease is a neglected tropical disease caused by the protozoan parasite *Trypanosoma cruzi*, which currently affects around 8 million people worldwide. The therapeutic arsenal against *T. cruzi* is so far limited to only two approved drugs, benznidazole and nifurtimox, that have considerable side effects and limited efficacy in the chronic stage of the disease. Here, we have resorted to supervised phenotypic machine learning models to explore drug repurposing opportunities and identify potential new therapeutic solutions for Chagas disease. **Methods**: More than 100,000 bioactivity data points were retrieved from ChEMBL and carefully curated according to the data-centric machine learning paradigm. After curation, two datasets comprising 344 compounds tested against *T. cruzi* Y strain trypomastigotes and 785 compounds tested against Tulahuen strain amastigotes were obtained and used to infer ensemble learning models with excellent average and early enrichment metrics in retrospective screening experiments (AUROC > 0.96 and EF_0.01_ > 58). A prospective screening campaign was then performed on DrugBank and the Drug Repurposing Hub databases, submitting eight in silico hits for experimental confirmation. **Results:** Six of the in silico hits confirmed their predicted trypanocidal effects. **Conclusions**: We have built portable meta-classifiers capable of identifying small molecules with trypanocidal activity against amastigotes, the clinically most relevant stage of *T. cruzi*. The predictive ability of this meta-classifier was experimentally validated.

## 1. Introduction

Chagas disease is a neglected infectious disease caused by the kinetoplastid *Trypanosoma cruzi*, historically endemic to Latin America. Currently, however, increasing prevalence was observed in previously non-endemic settings, because of rural-to-urban and international migration processes, affecting 6–8 million people worldwide [1]. There are only two drugs available for Chagas disease treatment: benznidazole (BZN) and nifurtimox. Both require prolonged treatments (up to 60 days), are associated with adverse events whose prevalence and severity increase with age, and their effectiveness in the chronic phase of the disease continues to be a matter of debate [2]. Despite this, the current number of compounds reaching the clinical stage has remained low [3].

Machine learning (ML) is a powerful data science approach that can assist data-informed decision-making solutions [4]. ML has been increasingly integrated into both ligand- and structure-based virtual screening approaches, to identify novel bioactive scaffolds with a desired pharmacological profile [5]. Although ML has historically been used in the context of target-based drug discovery (as a kind of in silico surrogate for in vitro assays), the current progress in artificial intelligence techniques and the availability of abundant systemic information (e.g., omics or phenotypic data) have increased the interest in using supervised ML tools to develop models that predict multiple and/or phenotypic responses [6,7,8,9].

Historically, ML applications in the field of drug discovery—and particularly in ligand-based approaches such as the QSAR paradigm—have relied on supervised, model-centric strategies (i.e., the use of labeled data and the development of new algorithms and models). Moreover, they have generally been grounded in the assumption that models trained and validated on larger datasets would exhibit improved generalization performance. However, recent trends—both in the broader ML community and in domain-specific pharmaceutical applications—challenge this view, noting that prioritizing data quality and the selection of appropriate representations, rather than simply increasing data volume or relying on more flexible models, can often lead to increased accuracy and optimized computational cost. Strategies such as active learning, semi-supervised learning, and operations such as data-quality assessment and data cleaning are concrete embodiments of this data-oriented paradigm [10,11,12].

Here, we have implemented a data-centric ML-based virtual screening approach to identify chemical compounds with activity against the clinically relevant stages of *T. cruzi*, i.e., trypomastigotes and amastigotes. Eight in silico hits were acquired and tested against both stages, with six of them confirming the predicted activity, thus validating the predictive power of our models.

## 2. Materials and Methods

### 2.1. Dataset Compilation, Curation, and Classification

**Figure 1:**
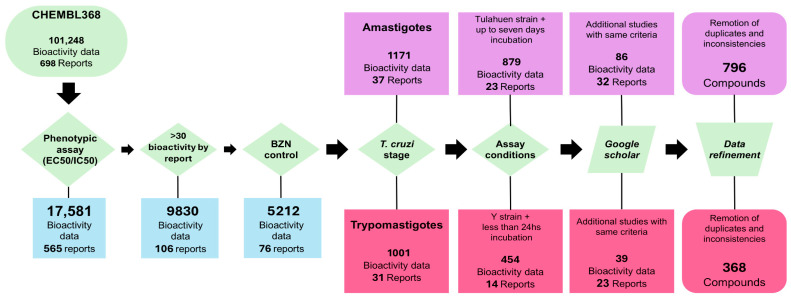
Dataset compilation and curation workflow.

We implemented a data-centric paradigm [10], which involves prioritizing data quality vs. data quantity, avoiding the inclusion of uncertain, noisy or mislabeled data. A general overview of the workflow is presented in Figure 1.

A bibliographic search was performed for small molecules tested against the two clinically relevant forms of *T. cruzi*: trypomastigotes (TRYP) and amastigotes (AMAS). The search began in the ChEMBL 34 database [13], filtering by organism (ChEMBL ID: CHEMBL368). We found more than 100,000 bioactivity records for *T. cruzi*, including both phenotypic and target-based assays. Bioactivity data against specific molecular targets such as enzymes or receptors were disregarded, and only phenotypic assays were retained. Initially, only the 17,581 bioactivity records corresponding to compound concentrations that inhibited 50% of parasite viability relative to the negative control (labeled as EC_50_ or IC_50_ in ChEMBL) were retained. To mitigate data noise associated with interlaboratory variability, only those reports that used BZN as a positive control and reported BZN EC_50_ were included.

Next, we curated the data separately for each clinically relevant stage of *T. cruzi*. In the case of TRYP, we selected only those studies where parasites were incubated with test compounds for 24 h or less, before quantification of parasite viability. To prevent noise related to inter-strain variability in drug sensitivity, only compounds tested against the Y strain (the most frequently represented in the dataset) were considered. In the case of AMAS, we selected those reports in which infected cells were incubated with compounds for up to seven days before amastigote quantification, and we retained only compounds tested against the Tulahuen strain. Fourteen reports met these criteria for TRYP [14,15,16,17,18,19,20,21,22,23,24,25,26,27] and 23 for AMAS [14,28,29,30,31,32,33,34,35,36,37,38,39,40,41,42,43,44,45,46,47,48]. Compounds with missing or inconsistent data were removed. Furthermore, we searched Google Scholar for additional studies meeting the same criteria as above and identified ten reports for TRYP [47,48,49,50,51,52,53,54,55,56] and nine for AMAS [57,58,59,60,61,62,63,64,65] (Figure 1).

**Figure 2:**
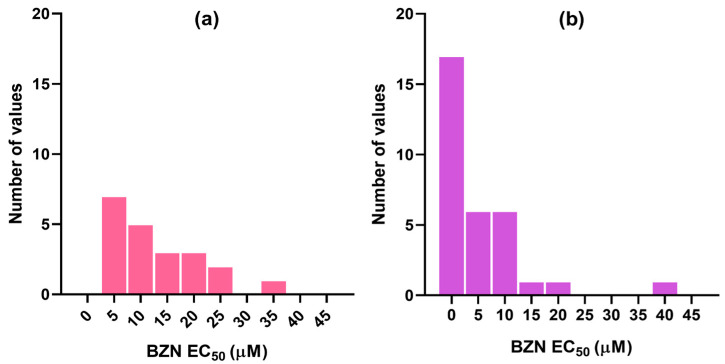
Distribution of BZN EC_50_s reported in the different studies from which the dataset compounds were retrieved for (**a**) trypomastigotes and (**b**) amastigotes.

It was observed that the positive control with BZN displayed variable results across studies (Figure 2). In order to mitigate noise due to interlaboratory variability, we classified the molecules as INHIBITORS or NON-INHIBITORS based on the ratio between their EC_50_s and BZN EC_50_. Molecules with EC_50_ values equal to or lower than that measured for BZN in the same study (i.e., EC_50_/BZN EC_50_ ≤ 1) were considered as INHIBITORS, while those with EC_50_/BZN EC ≥ 1.5 were classified as NON-INHIBITORS. Molecules with intermediate ratios (1 < EC_50_/BZN EC_50_ < 1.5) were excluded to mitigate data mislabeling due to assay uncertainty near the cutoff value.

The molecules in SMILES format were standardized using the super_parent function of the MolVS 0.1.1 package (https://molvs.readthedocs.io/en/latest/ (accessed on 1 November 2025)). Briefly, the largest organic fragment of each molecule was selected, the most common isotope of each atom type was assigned, charges were neutralized, and, since only conformation-independent descriptors would be considered for modeling purposes, stereochemical information was removed. After standardization, duplicate molecules were removed.

The final curated TRYP dataset consisted of 97 INHIBITORS and 244 NON-INHIBITORS, while the curated AMAS dataset consisted of 218 INHIBITORS and 577 NON-INHIBITORS.

### 2.2. Dataset Sampling

The dataset for each clinically relevant form of the parasite was representatively split into training and test sets to build and validate ML models. For this purpose, the dataset molecules were clustered using the iterative Random subspace Principal Component Analysis (iRaPCA) approach [66], which was applied independently to INHIBITORS and NON-INHIBITORS for each *T. cruzi* stage. By default, iRaPCA combines feature bagging of conformation-independent Mordred descriptors [67], dimensionality reduction using PCA, and clustering via the K-means algorithm. 100 subsets of 200 descriptors each were randomly sampled from a pool of 1613 descriptors. Features were normalized with the MinMaxScaler function of scikit-learn [68], and correlated descriptors (Pearson coefficient > 0.4) were removed. PCA was performed to obtain the first two principal components, followed by K-means clustering (varying K between 2 and 20). For each subset, 10 randomly picked seeds were tested, and the solution with the lowest within-cluster distance was retained. Clusters were evaluated using the Silhouette score [69], and the K value with the highest score was selected. Clusters containing more than 40% of molecules were subject to iterative clustering; smaller clusters were kept as they were.

From each INHIBITOR cluster, 70% of molecules were assigned to the training set. The set was balanced with an equal number of NON-INHIBITORS randomly selected from NON-INHIBITOR clusters. The remaining INHIBITORS and NON-INHIBITORS were used for external validation purposes. Table 1 summarizes the final dataset composition. Both datasets, labelled, have been included as Supplementary Materials

### 2.3. Model Generation and Validation

1613 conformation-independent molecular descriptors were computed for each dataset using Mordred [67]. Descriptors with missing values for any molecule and descriptors with low variance (<0.05) in the training set were disregarded. A total of 3000 random subsets of 200 descriptors each were generated. Within each random subset, highly correlated descriptors (Pearson coefficient > 0.85) were eliminated. In each subset, forward stepwise selection was applied to derive linear classifiers, allowing the inclusion of up to 13 descriptors for the TRYP models and up to 30 descriptors for the AMAS models (i.e., no more than one descriptor per 10 molecules in the training set).

The predictive power of each generated model was evaluated by calculating the area under the ROC curve (AUROC) in retrospective screening campaigns (see Section 2.4). Models were ranked according to their AUROC, and the descriptors included in each model were assessed. Starting from the top-ranked model, subsequent models were examined, and those sharing more than one descriptor with any higher-ranked model were discarded. For amastigotes, only 31 of the 3000 models remained, while for trypomastigotes, 99 of the 3000 models were retained. The difference arises because amastigote models incorporate a larger number of descriptors, which increases the chances of redundancy when selecting descriptors based on a feature bagging strategy.

Overall accuracy (Acc), balanced Acc, F-measure, precision, recall, and Matthews Correlation Coefficient (MCC) were computed for the test set. To enhance predictivity, models were combined using five operators: Minimum score (MIN), Average score (MEAN), score product (PROD), Average ranking (RANK), and voting (VOTE), as in previous studies [70,71]. AUROC and Boltzmann-enhanced discrimination of the ROC curve (BEDROC) were calculated for each ensemble. Internal validation was performed for those models included in the ensembles using Leave-Group-Out (LGO) cross-validation and Fisher randomization test. A total of 500 LGO rounds were conducted, in which 20% of the training set molecules were withheld in a stratified manner, and models were built with the remaining compounds. For Fisher randomization, 500 iterations were performed.

In previous studies from our group (aimed at diverse therapeutic indications, including antiparasitic, antiviral, and anticonvulsant agents), the general strategy of combining stochastic feature exploration with ensemble learning—occasionally complemented by target-structure-based approaches—has proven successful, with observed positive predictive values typically falling within the 30–100% range [70,71,72,73].

### 2.4. Retrospective Virtual Screening Experiments

In order to assess the ability of the models to identify active compounds in a scenario resembling a real virtual screening campaign (in which relatively few active compounds are expected to be scattered across the screened libraries among a relatively large number of inactive compounds), we carried out two retrospective screening experiments by seeding a small number of known active compounds (as shown in Table 1) among a large number of synthetic decoys generated by our in-house decoy generator LUDe [74].

It should be emphasized that synthetic decoys are usually generated to validate the enrichment capacity of virtual screening protocols focused on a specific target. To generate valid decoys, it is essential to assume that chemical compounds with molecular topologies very different from those of the known actives are unlikely to bind to the same binding site. In this case, since the phenotypic models generated are target-agnostic, it is possible that the compounds predicted as active may act through diverse mechanisms of action. For this reason, we not only checked the molecular dissimilarity of the decoys with respect to the active compounds against the strains of interest, but also against active compounds reported for other *T. cruzi* strains recovered from various bibliographic sources [33,36,40,41,42,43,44,45,75,76,77,78]. Our fundamental hypothesis at this point is that if the decoys display low similarity to a broad set of active compounds that are likely to interact with diverse targets, they are unlikely to be phenotypically active.

### 2.5. Prospective Virtual Screening

The model ensemble with the best early enrichment metrics for each parasite form was used for the prospective virtual screening of the DrugBank 5.1.6 database [79] and the Drug Repurposing Hub [80]. The compounds from these databases were standardized in the same way as previously described under Section 2.1. The score cutoff value was selected so as to optimize both the PPV surfaces [81] and the metrics obtained in the test sets.

To verify whether each screened compound belonged to the applicability domain of the model, we applied the leverage rule with a critical value of 3d/n (where d is the number of descriptors in each model and n is the number of compounds in the training set).

### 2.6. Biological Assays

For the evaluation of the trypanocidal effect of selected drugs on trypomastigotes, assays were performed using culture-derived trypomastigotes of the *T. cruzi* Y and K98 strains. Parasites were incubated in 96-well plates at a density ranging from 5 × 10^5^ to 1 × 10^6^ trypomastigotes per well, in a final volume of 100 μL of MEM supplemented with 5% serum. Test compounds were previously dissolved in dimethyl sulfoxide (DMSO) and added to the wells so that the final DMSO concentration was 1%. Wells containing 1% DMSO were used as vehicle controls, and 10 μM BZN served as a positive control. In the case of the Y strain, after 24 h of incubation at 37 °C, 10 μL of resazurin solution was added to reach a final concentration of 0.01 mg/mL. Plates were incubated for approximately 4 h at 37 °C to allow fluorescence development, and readings were performed in a microplate reader with excitation and emission wavelengths of 530 nm and 590 nm, respectively. To calculate EC_50_ in trypomastigotes, six points were used in a concentration range of 5 to 100 μM. At least three independent assays were performed. In the case of the K98 strain, after 24 h of incubation, motile parasites were counted in a hemocytometer chamber under a light microscope. Controls consisted of RPMI-1640 supplemented with 5% FBS as well as RPMI-1640 with 0.1% DMSO. At least three independent assays were performed.

To evaluate the trypanocidal effect of the selected compounds on intracellular amastigotes, the drug screening method reported by Buckner et al. was adapted. Vero cells were seeded at 1 × 10^4^ per well on a 96-well culture plate in 100 μL MEM-5% FBS. Tulahuen trypomastigotes overexpressing the *E. coli* β-galactosidase protein (LacZ clone C4 strain—ATCC^®^ PRA-330™, ATCC, Manassas, VA, USA) were used to infect Vero cell monolayers (MOI: 10:1) in triplicate. After 2 h incubation at 37 °C, the culture media were removed and non-internalized parasites were eliminated by washing each well with at least 10 vol of sterile PBS 1X, after which fresh MEM-5% FBS was added. After 16–18 h, the culture medium was replaced with culture medium bearing the compounds to be tested at the desired concentration. Test compounds were prepared in DMSO, ensuring a final DMSO concentration of 1%. Cells treated with 1% DMSO were used as vehicle controls. To determine the IC50, each drug was evaluated in at least six different concentrations (usually 0.78, 1.56, 3.13, 6.25, 12.5, and 50.0 μM). The plate was further incubated for another 72 h at 37 °C, after which the culture media were removed and cells and intracellular amastigotes were lysed in 100 μL lysis buffer (25 mM Tris pH 8, 2 mM EDTA, 2 mM DTT, 1% Triton X-100, 10% glycerol in ultrapure MQ water) for 10 min at 37 °C. Then, 100 μL 2X reaction buffer (200 mM sodium phosphate, pH 8, 2 mM MgCl_2_, 100 mM 2-mercaptoethanol, and 1.33 mg.mL^−1^ o-nitrophenyl-β-galactoside (ONPG)) was added, and the reaction was allowed to proceed until a yellow color developed (1–2 h at 37 °C). The absorbance at 420 nm was measured in a Synergy HTX multi-mode microplate reader (Biotek Instruments, Winooski, VT, USA) and normalized to the value obtained for the infection in the absence of inhibitors. IC_50_ for BZN was also evaluated following this method (concentrations evaluated: 0.10, 0.20, 0.39, 0.78, 1.56, 3.13, 6.25, 12.5, and 50.0 μM). Uninfected cells were included as blanks. Each compound was tested in at least two independent experiments.

Statistical analyses were carried out using GraphPad Prism 5.0, San Diego, CA, USA (www.graphpad.com).

## 3. Results

### 3.1. Trypomastigote Models

Using a random subspace approximation combined with a Forward Stepwise procedure, 3000 linear classifiers were obtained, which were reduced to 99 after excluding those with more than one redundant molecular descriptor. The performances of the four best models, including their behavior in the LGO cross-validation and randomization tests, are summarized in Table 2. Whereas the models showed suboptimal accuracy, the results of the LGO cross-validation and randomization tests suggest, respectively, that they are robust and not overfitted (note the similarity between the mean Acc in the LGO cross-validation rounds and the Acc obtained in the training set), and that the chance of spurious correlations is low (the mean Acc in the randomization test is in all cases of about 0.5).

The equations of the best-performing individual models are given as follows (Mordred’s descriptor nomenclature has been retained. Further details on these descriptors can be found at https://mordred-descriptor.github.io/documentation/master/descriptors.html (accessed on 1 November 2025)):

Model 583

Score = 2.865 − 0.548 GATS1p − 0.018 ATSC4d − 0.513 nFaHRing + 0.183 piPC6 − 0.305 NaasN + 0.354 Lipinski + 0.167 nS − 0.023 AATS1i

Model 2264

Score = −3.626 + 0.513 nFARing + 0.256 SaaS − 0.177 SaasN − 0.272 nFHRing + 0.243 BCUTv-1l + 0.060 AATS3dv + 0.083 NsOH

Model 595

Score = 0.217 − 0.022 SMR_VSA6 + 0.023 ATSC0p + 0.00021 ATSC7v + 0.017 EState_VSA2 − 0.129 ATSC2are + 0.277 NaaS − 0.033 SsF

Model 2977

Score = 2.351 − 0.031 AATS2i − 0.020 PEOE_VSA3 + 0.044 SRW09 + 0.297 AATS3s − 0.289 AATS5pe + 0.234 BCUTv-1l − 0.010 ATSC4d

The models reveal a relative abundance of 2D autocorrelations, including Geary autocorrelations (GATS1p), Moreau–Broto autocorrelations (ATSC4d, ATSC0p, ATSC7v, ATSC2are, ATSC4d), and averaged Moreau–Broto autocorrelations (AATS1i, AATS3dv, AATS2i, AATS3s, AATS5pe). These autocorrelations are weighted by different atomic properties, including polarizability (p), ionization potential (i), Allred–Rochow electronegativity (are), Pauling electronegativity (pe), van der Waals volume (v), among others, and reflect the distribution of such properties within the molecule, as well as whether a given distribution is favorable or unfavorable to trypanocidal activity. To some extent, these descriptors are linked to pharmacophoric features, although they only account for topological distances between atoms rather than their relative geometric coordinates. The nFARing descriptor suggests that aliphatic fused rings are favorable to trypanocidal activity, whereas nFHRing and nFaHRing indicate that aromatic fused hetero rings are unfavorable to such activity. The models also prominently incorporate several Kier–Hall Electrotopological State (E-state) descriptors, namely NaasN, SaaS, NsOH, NaaS, and SsF, which capture the contribution of specific atom types to biological activity (e.g., nitrogen atoms in amines bonded to two aromatic rings, sulfur atoms bonded to two aromatic rings, hydroxyl groups, and fluorine atoms).

**Figure 3:**
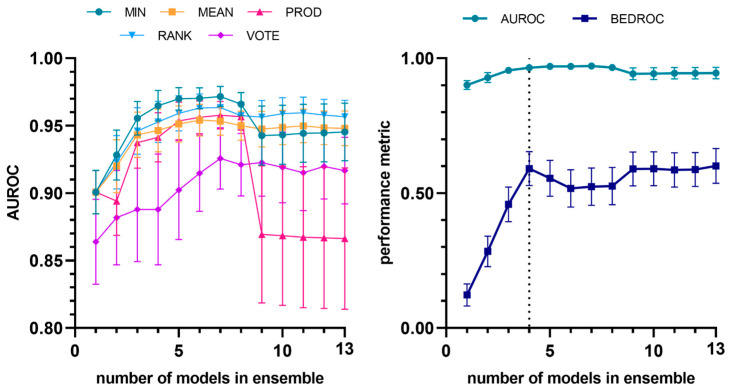
Evolution of AUROC vs. number of models (to identify trypanocidal compounds against trypomastigotes) incorporated into the model ensemble, considering different combination schemes (**left**). Evolution of average and early enrichment metrics (AUROC and BEDROC) vs. the number of models in the ensemble for the best-performing combination strategy (MIN operator) (**right**). The individual models are integrated into the ensemble according to their performance ranking in the first retrospective screening. The selected ensemble has been indicated with the vertical dashed line.

The selective combination of individual models (according to their performance in the first retrospective screening experiment) into meta-classifiers provided a significant improvement in terms of both average enrichment and early enrichment, as shown in Figure 3 and Table 3, where the chosen model combination is compared with the best-performing individual model. The ensemble built using the minimum operator consistently yielded the best results up to a combination of eight models. The highest value of the metric was observed when the best four individual models were combined (MIN-4 ensemble). It is worth noting that this constitutes a conservative way of combining the scores from individual models, since each compound is assigned the lowest score among those given by the models comprising the ensemble. Consequently, if even a single model within the ensemble predicts a compound as inactive, the compound will be classified as such, regardless of the predictions of the other models.

### 3.2. Amastigote Models

A strategy similar to that employed for deriving the trypomastigote models was used, combining feature bagging (3000 random subspaces) with Forward Stepwise selection. However, from those 3000 subspaces only 31 non-redundant models were derived in this case (i.e., models sharing no more than one molecular descriptor). This outcome was likely due to the larger number of training instances compared to the trypomastigote dataset, which allowed the inclusion of a greater maximum number of descriptors in the amastigote models, thereby increasing the likelihood of redundancy among models. Possibly also due to the larger number of training instances, some of the best-performing models (Table 4) achieved slightly higher accuracy than in the case of the trypomastigote models. The overall accuracy range in the training set was 0.756 to 0.813 for the top ten models. Similarly to what was observed for the trypomastigote models, the results of the internal validation studies suggest that the models are robust and arise from non-spurious correlations between the observed class and the selected molecular descriptors, with little to no evidence of overfitting.

**Figure 4:**
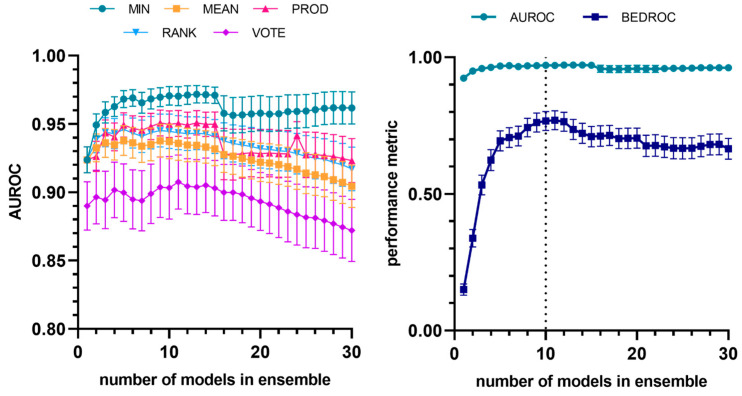
Evolution of AUROC vs. number of models (to identify trypanocidal compounds against amastigotes) incorporated into the model ensemble, considering different combination schemes (**left**). Evolution of average and early enrichment metrics (AUROC and BEDROC) vs. the number of models in the ensemble for the best-performing combination strategy (MIN operator). The dashed line corresponds to the selected ensemble (**right**). The individual models are integrated into the ensemble according to their performance ranking in the first retrospective screening.

Similar to what was observed for the amastigote models, the model ensemble achieved, in the retrospective screening experiments, an active enrichment capacity that was markedly higher than that obtained by the individual models. In this case, the combination of the ten best models using the MIN operator (MIN-10) yielded the best comparative performance (Figure 4). A comparison between the performance of the best individual model and that of the best ensemble is presented in Table 5. The equations of the ten models that comprise the best ensemble are provided as Supplementary Materials. Two-dimensional autocorrelations and E-state molecular descriptors remain the predominant descriptors in these models.

### 3.3. Prospective Virtual Screening

**Figure 5:**
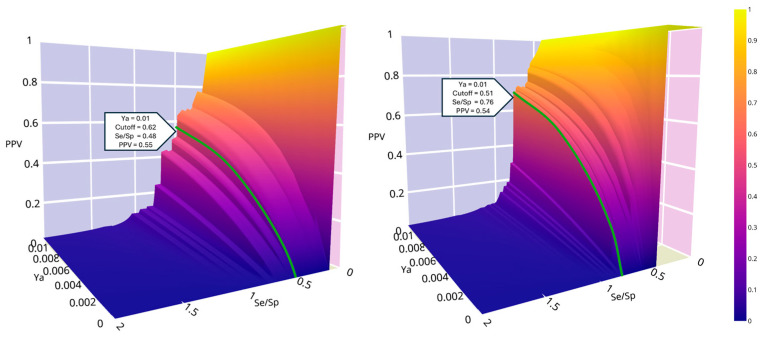
PPV surface plots for trypomastigotes (**left**) and amastigotes (**right**). Colorbar indicates the PPV values.

To establish the score cutoff value, we generated the PPV surface plots for trypomastigotes and amastigotes by applying MIN-4 and MIN-10 in the first retrospective experiment (Figure 5). For trypomastigotes, we set the cutoff score at 0.62. For amastigotes, the cutoff score value was set at 0.51. Using these thresholds, the estimated PPV for an assumed proportion of active compounds in the screened library of 0.001 the corresponding PPV value is 0.10, whereas for an assumed proportion of 0.01 the estimated PPV exceeds 0.5. In other words, in the worst-case scenario, 1 out of every 10 evaluated hits should be active.

For trypomastigotes, the MIN-4 ensemble was applied to the DrugBank and DRH databases. 125 molecules exceeded the cutoff value of the selected score, with 117 of them falling within the applicability domain of the model that provided the minimum score. Among them, 47 were approved drugs. For amastigotes, the MIN-10 ensemble was applied to the DrugBank and DRH databases. A total of 194 molecules exceeded the cutoff value of the selected score, of which 188 fell within the applicability domain of the model that provided the minimum score. Among these, 53 were approved drugs.

### 3.4. Experimental Confirmation of In Silico Predictions

Following the in silico predictions performed for each developmental stage of *Trypanosoma cruzi*, a total of eight candidate compounds were selected for experimental validation. Three of them—Altrenogest, Mifepristone, and Dienogest—were selected from the predictions based on the trypomastigote stage of the Y strain (DTUII). The remaining five—Cenicriviroc, Doramapimod, LSZ-102, Glesatinib, and Navitoclax—were obtained from predictions performed for the amastigote stage of the Tulahuen strain (DTUVI). All compounds were acquired from Cayman Chemical, except for LSZ-102, which was acquired from eMolecules.

#### 3.4.1. Preliminary Screening on Y Strain Trypomastigotes

The three compounds predicted to be active against trypomastigotes were initially tested at 10 µM and 50 µM on Y strain trypomastigotes obtained from infected Vero cell cultures. Among them, Mifepristone produced a 69.5% reduction in parasite viability at 50 µM, after 24 h of incubation, whereas Altrenogest and Dienogest did not exhibit detectable activity under the same conditions.

Attempts to determine the EC_50_ value for Mifepristone in this strain were unsuccessful, as the resulting dose–response curves did not provide a satisfactory fit, precluding reliable estimation of this parameter.

#### 3.4.2. Evaluation on K98 Strain Trypomastigotes

To further support these findings, the hit compounds were also evaluated against trypomastigotes of the K98 strain (TcI). In this assay, Mifepristone displayed measurable activity, with an EC_50_ value of 17.9 µM, confirming its trypanocidal potential. In contrast, Altrenogest and Dienogest remained inactive, consistent with the results obtained for the Y strain (Table 6).

#### 3.4.3. Evaluation of Compounds Predicted to Be Active Against Amastigotes

All compounds predicted in silico to be active against amastigotes of the Tulahuen strain displayed some degree of activity when tested on intracellular amastigotes infecting Vero cells. It is worth noting that both Navitoclax and Glesatinib also exhibited cytotoxic effects on Vero cells; accordingly, their estimated EC_50_s could not be determined reliably.

**Figure 6:**
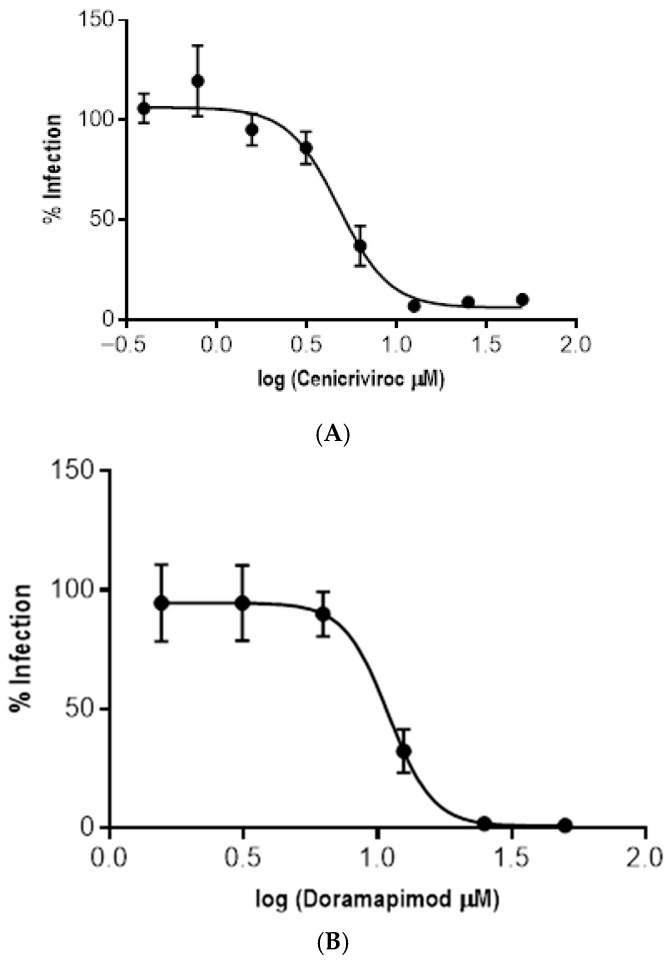

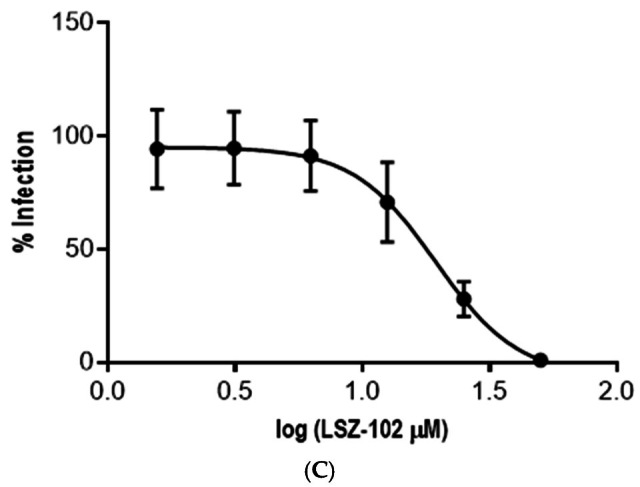
Dose–response curves for the three compounds active against *T. cruzi* amastigotes that did not present cytotoxic activity against Vero cells. (**A**) Cenicriviroc. (**B**) Doramapimod. (**C**) LSZ-102.

Among the remaining compounds, Cenicriviroc showed the strongest activity against intracellular amastigotes, with an EC_50_ value of 4.76 µM and a selectivity index greater than 10 (Figure 6 and Table 7).

## 4. Discussion

In this study, we developed machine learning models to predict the phenotypic activity of small molecules, separately for each clinically relevant stage of *T. cruzi*: trypomastigotes and amastigotes. Our approach presents some substantial differences compared with previously published studies based on Bayesian models [9,82].

First, in order to avoid introducing noise arising from inter-strain variability in drug sensitivity, we considered only bioactivity data obtained against specific *T. cruzi* strains (Y strain for the trypomastigote models and Tulahuen strain for the amastigote models; the selection of each strain was based on the relative abundance of high-quality data in the corresponding dataset).

Secondly, upon observing that the activity measured for one of the most frequently used positive controls, BZN, exhibits considerable variability across different studies and laboratories (see Figure 2 as an example), we decided that it would be worthwhile to establish a variable threshold—defined relative to the EC_50_ of BZN reported in the same study—to label a compound as active or inactive, instead of following the common practice of using a fixed cut-off value. We hypothesized that, in this way, we would compensate for the noise introduced by interlaboratory variability, by adjusting the cut-off value according to the activity measured for a common positive control.

In line with the aim of minimizing the incidence of potentially noisy data, we considered only compounds whose activity against *T. cruzi* had been quantitatively measured (i.e., reporting EC_50_ values) in studies where the EC_50_ of BZN had also been determined in parallel. Additionally, for data labeling, we introduced an activity gap—excluding compounds with 1 < EC_50_/BZN EC_50_ < 1.5—in order to minimize the impact of borderline cases and reduce the noise arising from measurements near the decision boundary between the two classes.

Judging by the performance of our models in both retrospective validation exercises and prospective in vitro validation, the data-centric strategy appears to have been successful. In the case of the best model ensembles, excellent metrics were obtained for both average and early enrichment, notably with AUCROC values above 0.9 in all retrospective screening campaigns, and exceeding 0.95 in three out of four cases (retrospective screening 1 for the tripomastigote MIN-4 model ensemble, and retrospective screening 1 and 2 for the amastigote MIN-10 model ensemble). It is also worth noting the diversity of scaffolds retrieved by our models.

**Figure 7:**
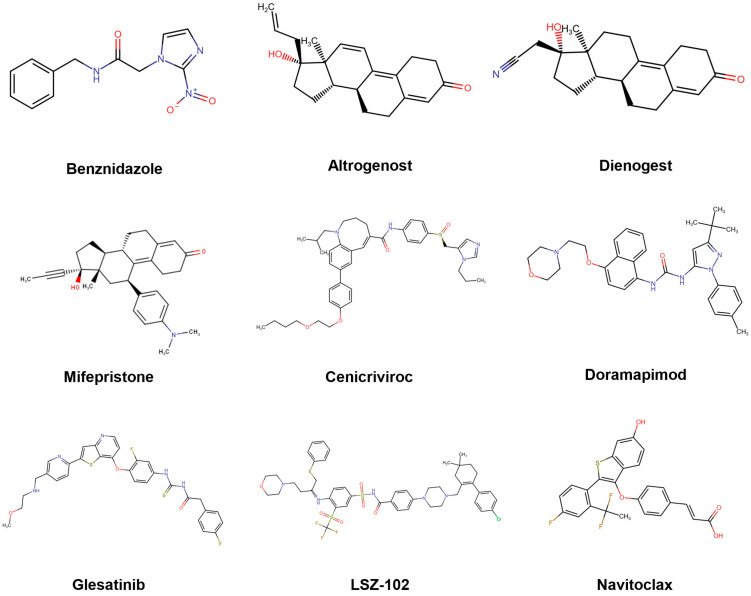
Molecular structures of the assayed in silico hits. BZN, the current first-line therapy, has been included for comparative purposes.

Since drug repurposing represents an accelerated strategy for the development of new therapeutic solutions—particularly appealing in the context of neglected diseases [83,84]—we decided to focus the initial prospective validation of our models on the screening of repurposing-oriented databases. Once validated, the models can now be applied to other, more translationally challenging compound collections. Possibly due to the relatively higher abundance of high-quality data, the predictive power of the amastigote model appears to be comparatively superior, considering the results summarized in Table 5 and highlighting the particular potential of cenicriviroc. It is also worth noting the diversity of bioactive scaffolds retrieved by our model ensembles (Figure 7). The molecular diversity of the confirmed hits is consistent with the fact that our models aimed at predicting phenotypic activity; therefore, the underlying mechanisms responsible for such activity may be multiple and/or heterogeneous. This would be consistent with the fact that the ensemble with best confirmed predictivity (the one that predicts activity against amastigotes) comprises ten individual models, and that the operator used to combine the individual classifier scores was the MIN operator, which assigns to each compound the score provided by the model yielding the lowest score among the ensemble members.

Having validated the predictive ability of these models, future efforts could be directed toward the in vivo evaluation of cenicriviroc, the acquisition of a larger number of in silico hits, and the virtual exploration of additional compound libraries. It is also worth emphasizing the portability of our models, whose descriptors were calculated using an open-access software package, thus allowing other research groups to directly and freely apply the models reported herein.

It should be noted that cenicriviroc is an experimental drug that acts by blocking the chemokine signaling receptors CCR2 and CCR5. It was originally developed as a potential treatment for HIV and later investigated for the management of liver diseases such as non-alcoholic steatohepatitis, due to its anti-inflammatory and antifibrotic properties, although a phase III clinical trial did not meet its primary efficacy endpoints for fibrosis [85]. This work therefore represents a potential initial step toward the rescue of a shelved drug.

## Figures and Tables

**Table 1:** Composition of the training and validation sets for each *T. cruzi* stage.

	TRYS				AMAS			
	Training	Test	Retrospective Validation 1	Retrospective Validation 2	Training	Test	Retrospective Validation 1	Retrospective Validation 2
Inhibitors	67	15	15	15	152	33	33	33
Non- inhibitors	67	177	-	-	152	425	-	-
Decoys	-	-	2318	2319	-	-	5312	5309

**Table 2:** Overall accuracy of the top-performing individual models on the training set, as well as their performance in the internal validation experiments (trypomastigote models). ^1^ Accuracies are presented as the average values of the metric ((across the internal validation rounds) ± its standard deviation.

Model	Accuracy	Accuracy LGO ^1^	Accuracy Fisher ^1^
583	0.769	0.732 ± 0.077	0.495 ± 0.105
2264	0.791	0.766 ± 0.074	0.497 ± 0.132
595	0.754	0.736 ± 0.077	0.499 ± 0.115
2977	0.791	0.760 ± 0.073	0.502 ± 0.116

**Table 3:** Statistical comparison of the best individual model to predict activity against *T. cruzi* trypomastigotes (Model 583) and the best model ensemble in the two retrospective screening experiments.

Model	Model 583		MIN-4	
	Retrospective Screening 1	Retrospective Screening 2	Retrospective Screening 1	Retrospective Screening 2
AUROC ^1^	0.898 ± 0.015	0.862 ± 0.023	0.963 ** ± 0.011	0.910 ** ± 0.023
BEDROC 100 ^1^	0.120 ± 0.044	0.104 ± 0.028	0.587 ** ± 0.065	0.451 ** ± 0.063
EF (0.01) ^1^	12.8 ± 5.4	3.3 ± 4.7	52.8 ** ± 7.6	44.0 ** ± 7.4

^1^ Represented by the average values of the metric ± its standard deviation (estimated by bootstrapping). ** *p* < 0.001.

**Table 4:** Overall accuracy of the top-performing individual models on the training set, as well as their performance in the internal validation experiments (amastigote models). ^1^ Accuracies are presented as the average values of the metric ((across the internal validation rounds) ± its standard deviation.

Model	Accuracy	Accuracy LGO ^1^	Accuracy Fisher ^1^
1487	0.832	0.813 ± 0.044	0.502 ± 0.079
351	0.829	0.806 ± 0.046	0.500 ± 0.094
1764	0.826	0.801 ± 0.048	0.500 ± 0.078
1860	0.816	0.799 ± 0.046	0.502 ± 0.082
283	0.819	0.799 ± 0.049	0.503 ± 0.083
313	0.816	0.801 ± 0.047	0.502 ± 0.096
1146	0.819	0.809 ± 0.046	0.500 ± 0.095
1609	0.796	0.786 ± 0.047	0.500 ± 0.080
723	0.776	0.756 ± 0.049	0.499 ± 0.092
2363	0.803	0.789 ± 0.047	0.501 ± 0.091

**Table 5:** Statistical comparison of the best individual model to predict activity against *T. cruzi* amastigotes (Model 1487) and the best model ensemble in the two retrospective screening experiments.

Model	Model 1487		MIN-10	
	Retrospective Screening 1	Retrospective Screening 2	Retrospective Screening 1	Retrospective Screening 2
AUROC ^1^	0.924 ± 0.010	0.919 ± 0.011	0.971 ** ± 0.007	0.960 ** ± 0.010
BEDROC 100 ^1^	0.144 ± 0.018	0.142 ± 0.019	0.769 ** ± 0.032	0.695 ** ± 0.034
EF (0.01) ^1^	8.8 ± 2.7	8.7 ± 2.5	74.1 ** ± 4.0	64.7 ** ± 3.6

^1^ Represented by the average values of the metric ± its standard deviation (estimated by bootstrapping). ** *p* < 0.001.

**Table 6:** Evaluation of the compounds predicted as active against trypomastigote forms against two different *T. cruzi* strains.

Compound	Activity on Y Trypomastigotes	EC_50_ on K98 Trypomastigotes (µM)
Altrenogest	Not active	Not active
Mifepristone	Active	17.9
Dienogest	Not active	Not active

**Table 7:** Evaluation of the compounds predicted as active against amastigote forms against Tulahuen strain and against Vero cells. Not toxic indicates that no toxic effect was observed up to 50 µM.

Compound	Activity on Tul Amastigotes (µM)	CC_50_ Vero Cells (µM)
Cenicriviroc	4.76	Not toxic
Doramapimod	10.97	Not toxic
LSZ-102	19.37	Not toxic
Glesatinib	6.49 *	~25
Navitoclax	2.14 *	6.03

* Indicates that these values are not reliable because of the simultaneous toxic effect against Vero cells.

## Data Availability

The datasets used to train our models have been made available as Supplementary Materials. Model building and validations, including dataset sampling, molecular descriptor calculation, and decoy generation have been realized using available open-source tools.

## Supplementary Materials

The following supporting information can be downloaded at: https://www.mdpi.com/article/10.3390/pharmaceutics17121513/s1, Supplementary_Material.docx: equations of the ten best models against amastigotes. Labelled datasets have been shared as Labelled_Datasets.xls.

## Abbreviations

The following abbreviations are used in this manuscript:

BZN	Benznidazole
ML	Machine learning
Acc	Accuracy
AMAS	Amastigotes
AUPR	Area under the precision-recall curve
AUROC	Area under the receiver operating characteristic curve
DMSO	Dimethyl sulfoxide
DRH	The drug repurposing Hub
EC_50_	Compound concentrations inhibiting 50% of parasite viability relative to control
LGO	Leave-Group-Out
MCC	Matthews correlation coefficient
MEAN	Average score
MIN	Minimum score
PCA	Principal component Analysis
PPV	Positive Predictive Value
PROD	Score product
RANK	Average ranking
*T. cruzi*	*Trypanosoma cruzi*
TRYP	Trypomastigotes
VOTE	Voting score
