# Supplementary material for: Application of Data-Centric Supervised Machine Learning to Predict Phenotypic Activity Against Clinically Relevant Stages of Trypanosoma cruzi
Source: Pharmaceutics. 2025 Nov 23;17(12):1513. doi: 10.3390/pharmaceutics17121513 (PMC12736270; doi:10.3390/pharmaceutics17121513)
Supplement: Supplementary file 1 [file pharmaceutics-17-01513-s001.zip › Supplementary_Material.pdf]

Supplementary_Material

Equations of the 10 best-performing individual models for amastigotes. Further details on these descriptors can be found at:

https://mordred-descriptor.github.io/documentation/master/descriptors.html):

Model 1487

Score= 0.119 - 0.670 SddsN + 0.014 BalabanJ + 0.147 SdssC + 0.023 AATSC3v - 0.023 SaaN + 0.239 NaaO - 0.308 GATS5s - 0.021 SsNH2 - 0.286 AATS2d + 3.53E-6 VR1_A - 0.025 ZMIC5 + 0.361 AATSC2i + 0.213 naRing - 0.007 AATS5v - 0.100 BCUTdv-1l - 0.020 SMR_VSA2 - 0.001 ATSC7dv + 0.004 AATS0v

Model 351

Score = -1.818 - 1.050 SddsN + 0.439 IC4 + 0.007 PEOE_VSA8 - 0.151 NdO - 0.094 NaaN - 0.028 VSA_EState4 + 7.91E-5 VR2_A - 9.4E-4 ATSC8dv + 0.082 nFHRing - 0.029 SaaO - 0.012 AATSC4v

Model 1764

Score: -0.419 + 0.468 NddsN - 0.175 AATS4s - 0.039 BCUTi-1h + 0.178 piPC4 - 0.001 ATSC4i - 0.379 CIC5 - 0242 NsNH2 - 0.278 AATSC1dv + 0.007 AATSC6m + 0.381 AATSC2i + 0.005 EState_VSA7 - 0.027 ATSC3p - 0.064 VSA_EState5 + 0.008 SMR_VSA6 + 0.027 SsCH3

Model 1860

Score = -1.204 + 0.448 NddsN + 0.399 IC5 - 0.047 GATS5se + 0.036 ATSC2p - 0.006 EState_VSA2 - 0.044 VSA_EState4 - 0.016 MID_O - 0.029 PEOE_VSA13 + 0.017 C2SP2 + 0.133 n9FRing + 0.0004 ATSC6Z + 0.064 GATS4se

Model 283

Score = -4.971 + 0.496 IC3 + 0.214 ATSC1pe – 0.002 SsF - 0.032 PEOE_VSA1 + 0.181 n9FHRing - 0.301 GATS4s - 0.019 PEOE_VSA11 - 0.027 SaaN + 0.511 AATS2are + 0.081 SsssN - 0.170 nAHRing + 0.301 GATS2se

Model 313

Score = -4.093 + 0.360 NddsN + 0.523 IC3 - 0.007 TopoPSA(NO) + 0.042 MID_N - 0.251 GATS5s - 0.013 ATSC4p - 0.112 NaaN + 0.294 AATS1se + 0.044 nHBAcc

Model 1146

Score = 0.504 - 1.071 SddsN + 0.111 nFRing + 0.058 Diameter - 0.005 TopoPSA(NO) + 0.432 IC1 - 0.010 ATSC7d - 0.0002 ATSC4v - 0.006 EState_VSA2 - 0.163 ATSC1are - 0.274 AATS4se

Model 1609

Score = 0.019 - 0.318 GATS3s + 0.012 TSRW10 + 0.007 ATSC3i - 0.277 GATS5s + 0.016 ATSC1pe + 0.074 NsCl - 0.031 VSA_EState4 + 0.019 SaaCH + 0.161 NdsCH - 0.124 NdssC + 0.050 C3SP2 - 0.427 AATS4d + 0.410 IC1 - 0.111 naHRing

Model 723

Score = 2.859 - 0.683 BalabanJ - 0.528 GATS4s - 0.084 ATSC4pe - 0.236 VE1_A + 0.157 ETA_beta_ns_d - 0.004 SMR_VSA9 + 0.006 AATSC6m + 0.141 nFRing - 0.048 C1SP2

Model 2363

Score = -1.577 + 0.111 SaasN + 0.092 SLogP + 0.050 BCUTs-1h + 0.381 IC2 - 0.018 MDEC-33 - 0.043 SaaO + 0.033 AATSC6Z - 0.006 SlogP_VSA5 - 0.015 PEOE_VSA3 - 0.066 C1SP2 + 0.280 Xch-7d
